# Scoping review of the current landscape of AI-based applications in clinical trials

**DOI:** 10.3389/fpubh.2022.949377

**Published:** 2022-08-12

**Authors:** Fidelia Cascini, Flavia Beccia, Francesco Andrea Causio, Andriy Melnyk, Andrea Zaino, Walter Ricciardi

**Affiliations:** Section of Hygiene, Department of Life Sciences and Public Health, Università Cattolica Del Sacro Cuore, Rome, Italy

**Keywords:** artificial intelligence, clinical trials, patient recruitment, trial design, scoping review

## Abstract

**Background:**

Clinical trials are essential for bringing new drugs, technologies and procedures to the market and clinical practice. Considering the design and the four-phase development, only 10% of them complete the entire process, partly due to the increasing costs and complexity of clinical trials. This low completion rate has a huge negative impact in terms of population health, quality of care and health economics and sustainability. Automating some of the process' tasks with artificial intelligence (AI) tools could optimize some of the most burdensome ones, like patient selection, matching and enrollment; better patient selection could also reduce harmful treatment side effects. Although the pharmaceutical industry is embracing artificial AI tools, there is little evidence in the literature of their application in clinical trials.

**Methods:**

To address this issue, we performed a scoping review. Following the PRISMA-ScR guidelines, we performed a search on PubMed for articles on the implementation of AI in the development of clinical trials.

**Results:**

The search yielded 772 articles, of which 15 were included. The articles were published between 2019 and 2022 and the results were presented descriptively. About half of the studies addressed the topic of patient recruitment; 12 articles reported specific examples of AI applications; five studies presented a quantitative estimate of the effectiveness of these tools.

**Conclusion:**

All studies present encouraging results on the implementation of AI-based applications to the development of clinical trials. AI-based applications have a lot of potential, but more studies are needed to validate these tools and facilitate their adoption.

## Highlights

- The use of AI in the design and conduct phases of a clinical trial has a positive impact in terms of efficacy, safety and cost containment.
- Aiding the translation process from bench to bedside, through coordinated policies and funding, would promote public health and quality of care.
- Artificial intelligence is far from being widely adopted in clinical trials, but it is highly promising for transforming clinical development.
- More studies are needed to validate AI-based tools and facilitate their adoption.
- Public health policies could promote AI adoption while ensuring standardization and quality.

## Introduction

Clinical trials are pivotal for discovering and translating new treatments for diseases, as well and ways to detect, diagnose, and reduce the chance of developing the disease to the market. Their importance lies in the fact that they can show what does and does not work in humans that cannot be learned in the laboratory or in animals thus allowing them to produce the highest level of evidence quality. Nonetheless, their increasing complexity makes trial design, execution and completion challenging (1).

It has been estimated that between 33.6 and 52.4% of all Phase I, II and III drug development clinical trials fail to proceed to the next trial phase, leading to a 13.8% overall chance that a drug tested in Phase I reaches approval. If the trial design phase is also taken into account, this percentage drops to around 10% (2). This has an enormous impact on drug development cost: after accounting for failed trials, the estimated average cost of research and development required to bring a drug to market is $1.3 billion (3). Fueled by the rapidly increasing amounts of medical data that are available to researchers, including those provided by Electronic Health Records (EHRs) and wearable devices, sophisticated machine learning and deep learning algorithms have the potential to save billions of dollars, speed up medical advances and expand access to experimental treatments (4).

Patient recruitment is one of the most challenging steps in clinical trials but by applying artificial intelligence (AI), researchers could significantly improve efficacy and safety while reducing costs. About one in every five clinical trials does not complete its enrollment of participants mostly due to the complex, stringent, and rigid inclusion criteria researchers must apply and adhere to (5). Challenges in subject enrollment and matching cause extension of enrollment deadlines delay the submission of the trial protocols for regulatory approvals and subsequently cause a deferment of the product launch beyond the initially planned dates. Additionally, selection bias can lead to results that are not generalizable because populations that were underrepresented may not respond well to the intervention (6).

Despite these issues related to patient eligibility and enrollment, a 2017 study stated that 75% of patients reported that they would be “somewhat” (44%) or “very” (31%) willing to participate in a clinical trial. However, only 3%−5% of the oncology patients identified in the study are matched into and enrolled in a clinical trial (7, 8).

In addition, improved selection of specific patient populations for trials may decrease the sample size required to observe a significant effect, reducing the number of patients that are unnecessarily exposed to harmful treatment side effects as well as costs related to the trial (9).

Considering the complexity of clinical trial development, AI seems to offer an innovative solution. Predictive models based on AI are already widespread in the healthcare field and AI contributes to data extraction, analysis and elaboration for those models. The use of AI to improve the data-driven approach in clinical pathway design and implementation provides important evidence for its potential application in clinical trial design (10). Thus, to improve clinical trials, researchers in academia and the pharmaceutical industry are turning to various types of artificial intelligence, such as machine learning, deep learning and natural language processing.

However, there is still scarce evidence of AI applications in clinical trials in scientific literature. We performed a scoping review to address this gap, trying to provide an overview of the most relevant applications of AI-based tools in clinical trial design and the conduction of the first three phases, focusing on patient recruitment. Specifically, we wanted to highlight the most relevant aspects of clinical trial design affected by this innovation and the effectiveness of such AI tools and their effect on clinical trial success. In addition, we aimed to identify practical examples of AI applications.

## Methods

In order to serve our objective, we conducted a literature review following the guidelines of Preferred Reporting Items for Systematic Reviews and Meta-Analyses Extension for Scoping Reviews (PRISMA-ScR) (11). We adopted the specific methodology of scoping review in order to provide a map of key concepts on the current landscape of AI-based applications in clinical trials, complying with the PRISMA-ScR checklist, reported as Supplementary Appendix 1 (12, 13). We believe a scoping review could prove to be ideal for this study, considering the wide range of aspects of the explored topic and the emerging evidence from a range of study designs, underpinning the main sources and types of evidence available. The need to make a timely assessment also lies in recognizing the topicality and perpetual evolutions of the issue and thus the need to draw drivers for action and future research from the scientific literature. We performed a literature search of PubMed database and retrieved articles published in English that were published up to June 2022 and addressed artificial intelligence and clinical trials. All years were considered. We used the following search query: *(“Clinical Trials as Topic”[Mesh]) AND “Artificial Intelligence”[Mesh]*.

We adopted the following definition of AI, as issued by the Council on Artificial Intelligence of the OECD: “An AI system is a machine-based system that can, for a given set of human-defined objectives, make predictions, recommendations, or decisions influencing real or virtual environments. AI systems are designed to operate with varying levels of autonomy.” (14).

We did not apply restrictions by article type. Articles contemplating artificial intelligence as an intervention in the trial were excluded. For reasons of feasibility, we restricted this study to only the first three phases of clinical trials, excluding the fourth phase of pharmacovigilance and pharmacoepidemiology and including the clinical trials design phase and the protocol development instead. This choice was motivated by the greater potential impact of AI in the phases considered (15, 16). Additionally, to retrieve further pertinent publications, we did backward reference searching considering the eligible articles. Four reviewers independently screened literature search results and extracted data from included studies. Any discrepancy was solved by discussion between the researchers. The extracted data was reported in an excel sheet and included: author, year, article type, clinical trial phase, use of AI. A narrative approach was used to synthesize the extracted data, providing a descriptive summary of each included article, and categorizing the results between studies that did and did not provide quantitative results.

## Results

**Figure 1:**
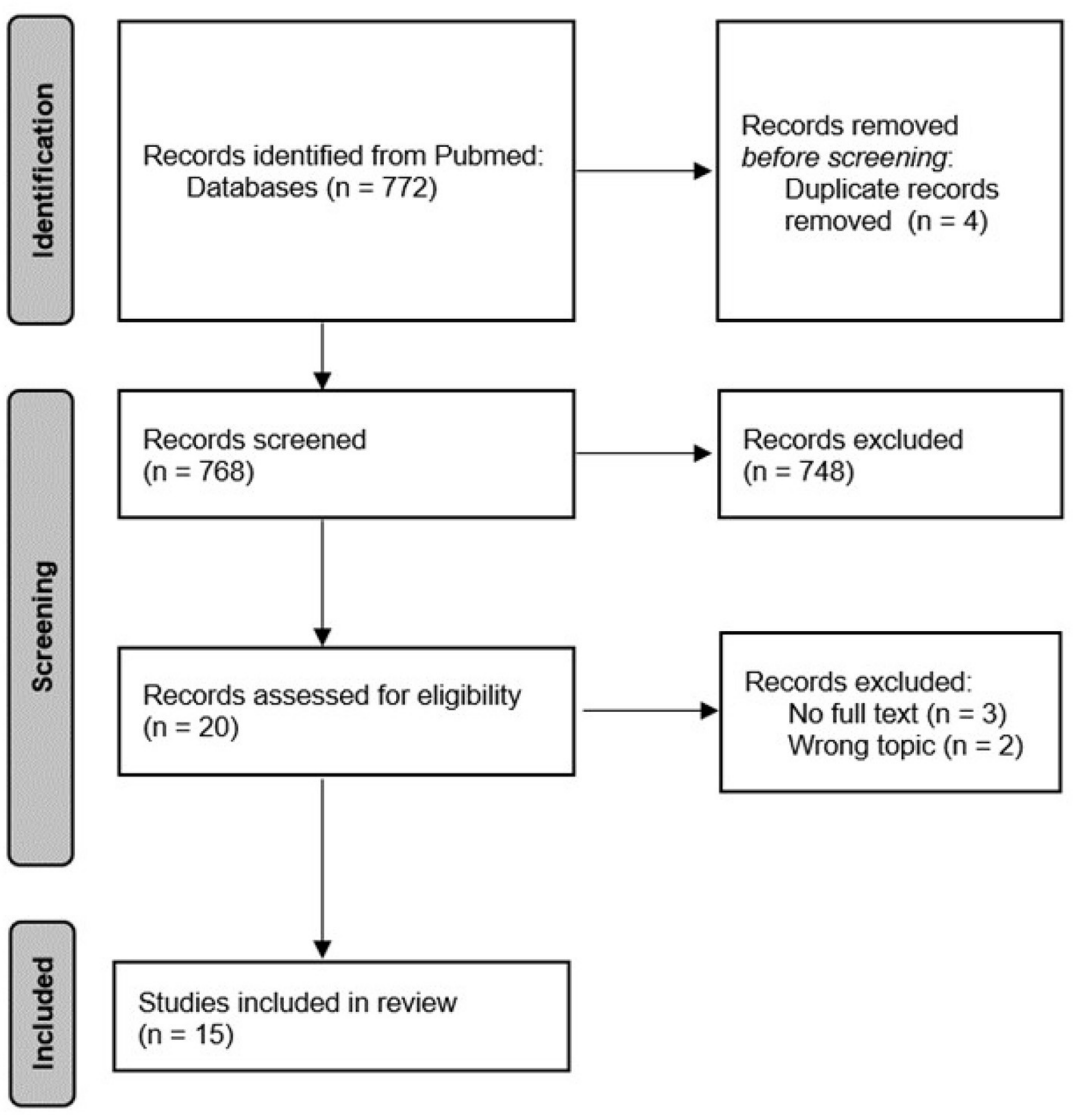
Flowchart of the search strategy.

The search query yielded 772 articles. After removing the duplicates, we screened titles and abstracts for 768 articles. Twenty records met the eligibility criteria and had the full text was evaluated. Subsequently, three articles were excluded because the full text was not available, and two articles were excluded because the topic addressed was not in line with the inclusion criteria. Finally, we included 15 articles. The flowchart of the search strategy is reported in Figure 1.

The included articles were published between 2019 and 2022, and were commentary (4, 17–19), reviews (20–24), and observational studies (5, 25–29). Almost half of the studies (47%) (4, 5, 19, 24, 25, 27, 29) hinged on patient eligibility and recruitment for clinical trials. An overview of our results, focusing on the main topics presented, is reported in Table 1.

**Table 1:** Included articles overview.

**Author, [REF]**	**Year**	**Article type**	**Main topics**	**Examples of AI application**	**Main findings for observational studies**
Harrer et al. (24)	2019	Review	patient cohort selection, recruiting techniques, monitoring of patients during trials	CTS tool, NPL-based algorithm	
Ngayua et al. (20)	2020	Review	AI adoption in clinical trials		
Delso et al. (23)	2021	Review	Patient recruitment and follow-up		
Hariry et al. (22)	2022	Review	AI-based tools for clinical trials	ML-based algorithm, TREWScore, DNN-based algorithm, AI Clinician	
Kolluri et al. (21)	2022	Review	AI-based tools for clinical trials' protocol design and oversight		
Krittanawong et al. (17)	2019	Commentary	Patient randomization and eligibility	IBM Watson, Mendel.AI	
Woo (4)	2019	Commentary	Patient recruitment	Criteria2Query, DQueST	
Weissler et al. (18)	2021	Commentary	Trial protocol design and development	Trials.AI, Mendel.AI, Deep6AI, AiCure	
Weng and Rogers (19)	2021	Commentary	Patient eligibility, medical record analysis	Trial Pathfinder	
Alexander et al. (26)	2020	Observational study	Patient eligibility and recruitment, AI-based tools for clinical trials	IBM Watson for CTM (proprietary and commercially focused)	95.7% accuracy for clinical trial exclusion and 91.6% accuracy for overall eligibility assessment compared to clinicians' assessment; however, clinician input and oversight were still required
Beck et al. (5)	2020	Observational study	patient recruitment and matching	IBM Watson for CTM (proprietary and commercially focused)	WCTM and manual review agreed on trial eligibility determinations in 81%-96% of patients. WTCM reduced time for screening by 78%
Calaprice-Whitty et al. (25)	2020	Observational study	patient eligibility, medical record analysis	Mendel.ai software (proprietary and commercially focused)	Increase in 24%−50% of correct patient identification for eligibility over standard practice
Vazquez et al. (29)	2020	Observational study	patient recruitment	ResearchMatch	Deep learning models had the highest likelihood of identifying patients that were potentially interested in participating in Clinical Trials
Haddad et al. (27)	2021	Observational study	patient eligibility, medical record analysis	AI-CDSS (IBM Watson, proprietary and commercially focused)	The overall accuracy of breast cancer trial eligibility determinations by the CDSS was 87.6% compared to manual screening
Yao et al. (28)	2021	Observational study	Patient recruitment, AI-based tools for clinical trials	Her NLP-based algorithm	Trial ongoing Clinicaltrials.gov: NCT04208971

ML, machine learning; NLP, natural processing language; DL, deep learning; AI, artificial intelligence; DNN, deep neural networks; EHR, electronic health records; CDSS, clinical decision support system; CTM, clinical trial matching; CTS, clinical trial simulation.

The use of AI to aid clinical trials has been recently addressed in a systematic review by Ngayua et al. This work showed an increase in research interest in this area over the last 10 years, with a third of all listed publications being from 2018 to 2020 (20).

Despite most publications being “overview” studies, some focused on specific aspects such as recruitment, eligibility, and patient matching. With a stricter focus on machine learning (ML), Weissler and colleagues wrote a commentary on the role of ML in clinical research, reporting the key areas of clinical trial methodology in which ML holds particular promise. Among these, they report a natural language processing (NLP)-based software to identify potential pitfalls and barriers to trial completion, though they emphasized that peer-review is lacking (18).

In another commentary, the functioning and the application of NLP is reported with the examples of two open-source platforms that assess patients' eligibility to participate in a clinical trial. The first platform, *Criteria2Query*, enables researchers and administrators to search databases without needing to know a database query language; whereas the second platform, *DQueST*, reads trials on ClinicalTrials.gov and then generates plain-English questions for eligibility surveys (4).

Similarly, Harrer et al. provided an overview of AI for clinical trials, focusing on patients' selection and monitoring. Specifically, trial eligibility has a heavy processing burden on doctors and patients, however, it is the key step in the patients' selection. The article proposes that the NLP tool should facilitate EHR screening. Machine learning empowers this system to learn and evolve. Assistive systems using these AI techniques or subsets could automatically analyze EHR and clinical trial eligibility databases, finding matches between specific patients and recruiting trials. In addition, AI and ML methods may also be used to dynamically predict the risk of patients' dropout (24).

More AI-based technologies are shown by Hariry and colleagues. They presented some of the attempts to predict patient outcomes in clinical research using ML, TREWScore, reinforcement learning agents, and deep neural networks. These AI methods were deployed for risk assessment, multitask predictions, disease modeling, and optimal decision making. The authors also highlighted the potential challenges and applications of embedded tools and devices in clinical trials and AI for a prospective Pharma 4.0 framework (22).

Weng et al. reported a software tool, called *Trial Pathfinder*, that uses EHR data to compare the survival outcomes of individuals who did or did not receive a certain drug treatment to assess the effects of including or omitting eligibility criteria from the original clinical trial (19). Using EHR data, Krittanawong et al. highlighted the potential for AI to guide adaptive clinical trials and produce higher-quality scientific evidence in cardiovascular medicine. In particular, they evaluated the applications of AI to operationalize and optimize study design and simulate data analysis rounds so they could improve the following: ongoing clinical trials, eligibility through EHR screening, predictive analytic ability and early negative result identification. A potential added benefit of this as well would be the possibility of reducing both the time and cost associated with more traditional trials (17).

Vazquez et al. assessed the use of supervised machine learning classifiers and a convolutional neural network (CNN) to analyze the interest in the participation of individuals in an online clinical trials registry. Deep learning was shown to be a promising approach in identifying individuals more likely to participate in a clinical trial and could further be used for recruitment resources to target those individuals more actively (29).

The benefits of nonparametric Bayesian learning as a tool in clinical trial design are presented in Kolluri et al.'s review. In oncology dose-finding trials, nonparametric Bayesian learning could offer efficient and effective dose selection (especially in those where patients having multiple types of cancers cause heterogeneity) (21).

Delso et al. described the capability of AI trials to boost patient recruitment, screening, enrollment, and monitoring, fostering patient empowerment and personalized approaches. AI can be directly applied to evaluate the performance of clinical trial pipelines to identify common characteristics associated with regulatory approval or refusal, including efficacy, safety issues and strategic and financial aspects. Moreover, using AI in clinical trial evaluations can reduce the impact of human error in data collection, discover trends, pinpoint relevant insights, and source quality information to optimize production, supply chain and logistics (23).

Five studies in our results reported the impact of AI-based tools in clinical trials in a quantitative manner, compared to human-based performances.

Calaprice-Whitty et al. retroactively tested an AI-powered technology on oncology studies protocols in order to evaluate its application in patient enrollment and then compare it to the results actually achieved in a typical setting, on the same data. The applied technology includes text recognition in scanned documents, natural language understanding of the clinical text and automated clinical reasoning. Two different protocols of an AI-powered technology resulted in an increase in the number of patients correctly identified as potentially eligible for clinical trial participation (24% and 50% increase respectively). In addition, they also demonstrated a significant reduction in the expected elapsed time between patient eligibility and identification for screening (25).

Xiaoxi Yao et al. showed another example of an AI-based algorithm used to improve patient recruitment, specifically by helping to predict which patients were more likely to have an undiagnosed disease by screening electronic medical records.

AI and ML can also be used on unstructured medical records with the help of NLP (28). Thaddeus Beck et al. and Alexander et al. provided an example of two automated clinical trial matching systems that extract patient and trial characteristics from unstructured sources and help match patients to clinical trials by applying NLP and ML. Agreement between the gold standard (manual selection by professionals) and the AI algorithms was very high, especially in eligibility determinations (81–94 and 91, respectively) (5, 26). On the same note, Haddad et al. evaluated the ability of an AI clinical decision support system (CDSS) to identify eligible patients for two groups of patients in a set of clinical trials by processing structured and unstructured patient data in the EHR to derive patient- and tumor-specific attributes. Its clinical performance was assessed using a manual review as the gold standard. The overall accuracy of breast cancer trial eligibility determinations by the CDSS was 87.6%. CDSS sensitivity was 81.1% and specificity was 89% (27).

## Discussion

Our results explored the applications of AI in clinical trials. Most of the included articles give an overview of the potential application of AI, providing examples of software and tools to improve patients' recruitment, matching and eligibility in trial design and Phases I–III. Only five studies had a quantitative estimate of the efficacy of AI in these critical phases of clinical trial design. All of the studies showed encouraging results, specifically regarding increased speed in the recruitment (25) and similarity of performance metrics to the gold standard (5, 25–27). Despite this, given the small sample size, a greater number of studies are needed to properly assess the efficacy and efficiency of AI use in clinical trial design.

While traditional double-blinded, randomized, controlled clinical trials remain the gold standard for biomedical evidence generation, augmentation with AI tools offers the potential to improve the success and efficiency of clinical research, increasing its positive impact on all stakeholders.

Even if Ngayua et al. tried to systematically address the topic, this is relatively a new field, and the publication period and the translational time (bench to bed) might hinder a wider adoption of AI (20). Therefore, developing guidelines and recommendations for this kind of AI application could overcome heterogeneity and facilitate the design and the conduction of clinical trials.

In 2020, the CONSORT-AI (Consolidated Standards of Reporting Trials-Artificial Intelligence) extension reported guidelines for clinical trials evaluating interventions with an AI component. It was developed in parallel with its companion statement for clinical trial protocols: SPIRIT-AI (Standard Protocol Items: Recommendations for Interventional Trials-Artificial Intelligence). This instrument tried to overcome the challenges of designing and reporting randomized clinical trials seeking to evaluate newer interventions based on, or including, an AI component (30).

However, scarce attention was paid to the adoption of AI-based tools for designing and conducting “classical” clinical trials. More specifically, improving patient recruitment, matching and eligibility in trial design and Phases I-III in clinical trials could make the entire process increase in effectiveness and safety, e.g., by more active targeting of individuals to be recruited, as pointed out in one of the studies in our results, as indicated by Vazquez et al. (29). As shown in Table 1, patient eligibility and recruitment are recognized as a crucial step in clinical trials, yet studies are not uniform in identifying a preferred solution or comparing different instruments. In fact, two of the key factors causing a clinical trial to be unsuccessful are patient cohort selection and recruiting mechanisms which fail to bring the best-suited patients to a trial in time, as well as a lack of technical infrastructure to cope with the complexity of running a trial in the absence of reliable and efficient adherence control, patient monitoring, and clinical endpoint detection systems. Harrer and colleagues brought to attention two relevant issues: suboptimal patient cohort selection and recruiting techniques, paired with the inability to monitor patients effectively during trials, are two of the main causes of high trial failure rates: only one of 10 compounds entering a clinical trial reaches the market (24). These steps also consume time and resources, as patient recruitment takes up one-third of the overall trial duration and Phase III trials carry 60% of the total costs for moving a drug through all trial phases.

However, there are still some factors that should be considered before implementing AI-based approaches in research. Haddad and colleagues underlined how the favorable ratio of cost-efficacy is indirectly assumed and additional research is needed to explore whether increased efficiency in patient recruitment and trial design translates to improvements in trial feasibility, assessment and cost (27). Transferability of results is also a limiting factor and could be addressed in future research (31).

AI-based applications have a lot of additional room for improvement. Machine learning algorithms rely on feeding a huge volume of training data from which they can learn but this requires time-consuming human mediation. This is a stumbling block in academia but could be overcome by industry. For example, software developed by Deep 6 AI was used by researchers at the Cedars-Sinai Smidt Heart Institute in California, to find 16 suitable participants for a trial in 1 h while a standard approach found 2 in 6 months. Similarly, in a pilot study, IBM's Watson for Clinical Trial Matching System increased the average monthly enrollment for breast cancer studies by 80% (4).

The validity and quality of this kind of instrument for broader applications still need to be further addressed. For example, IBM Watson resulted in an overall eligibility accuracy of 87.6% in the protocols where it was tested (32). However, to ensure results' reproducibility and clinical validity, AI-based models need to be evaluated and validated. Orienting future studies on this aspect could promote further adoption of AI in clinical trials, giving researchers and clinicians a wide range of possible interpretations for future uses at all levels of the health care system. At a micro-level, this includes patient screening and selection in clinical trials, as many of the included studies show, but also in several other fields, such as drug prescription adequacy (33). Moreover, AI and machine learning tools can increase flexibility and scalability for risk stratification, diagnosis and classification, and survival predictions. Some examples in oncology care include *CURATE.AI* for patient's dose-efficacy profile and *Augurium* for accuracy in oncology care (34). In addition, healthcare service delivery could benefit from the adoption of AI-based tools for patient administration, clinical decision support, patient monitoring and healthcare interventions, creating AI-augmented health systems (35). At the meso-level, artificial intelligence has shown to be significantly helpful when it comes to health service delivery, as seen in a review in the field of ophthalmology (36). At a macro-level, health systems have demonstrated a benefit from the implementation of AI in response to the COVID-19 pandemic, both in the first wave and in the following ones (10, 37). This is particularly relevant in reducing inequalities at all levels of the healthcare system (38). The clinical trial design and planning stage involves a wide range of services and stakeholders, producing a great, but fragmented, amount of data. The integration of all the key actors in a coordinated framework could be beneficial for a trial's success and ease the passage from one phase to another. Applying AI-based systems to the integration process would pave the way for Pharma 4.0, as extensively described in the work of Hariry et al. (22). Therefore, it is necessary to provide for collaborations among stakeholders, as well as allocate funds to promote research and pilot studies to validate AI-based applications. The involvement of public institutions and organizations, along with private ones, could overcome some implementation barriers related to public trust. Health professionals should be trained by updating degree programs and designing training courses to facilitate the adoption of digital tools. Digital literacy should be improved in order to translate the promising results of AI-based applications into clinical practice, particularly in the design and conduct of clinical trials.

This work has some limitations that should be mentioned. By using search terms only in the English language, we might have not been able to identify articles in the national languages of different countries. In addition, it could be possible that relevant articles were undetected by our search query, especially in the case of studies on specific applications of AI. Additionally, we performed the search only on PubMed but additional articles might have been available on other databases. Moreover, the high heterogeneity of the included studies, in terms of methodology and topics, and the lack of quantitative information in most of them, did not allow us to make an accurate comparison among them. Many studies have considered only certain aspects of the clinical trial process, providing qualitative considerations. It should be considered that any specific AI-based application should be validated and thoroughly evaluated, for both economic and ethical considerations, before being employed in practice. This significant gap in the literature, as can be inferred from the paucity of quantitative analysis in our results, is both a limitation of this scoping review and the reason we opted for this type of article.

Despite these limitations, our work presents some important points of strength. This is the first attempt to summarize AI applications in clinical trials in a comprehensive way. Even if a systematic review has been conducted on the topic (20), and is included in the results, this work proposes a different approach to this topic, summarizing results quantitively, rather than quantitatively; attempting to identify the underlying issues in this research field, and offering a starting point to breach the gaps in current consensus. Focusing on the possibilities and challenges of AI in healthcare is a prioritized strategy for implementing digital health in clinical care and contributing to the sustainability of healthcare systems while providing the best care for patients. Presenting the possible applications, and the successful use cases also offers fertile ground to policymakers and relevant stakeholders for addressing gaps and promoting AI uptake.

## Conclusions

The application of artificial intelligence in clinical trials is a new and promising field. Nevertheless, there is a lack of studies on the quantitative evaluation of the impact of AI tools in the design and conduction of a clinical trial. Optimizing the early stages of trials would save time and resources, with benefits in terms of sustainability and use of resources. The definition of guidelines and best practices could promote the use of artificial intelligence in research and trials and create integrated networks of research and development with the involvement of all relevant stakeholders. Our work responds to the gap in the literature and provides a comprehensive overview to define fields of action and future possibilities for collaborations between healthcare professionals, industry, citizens, patients and policymakers. The application of digital innovation can foster healthcare transformation and implement healthcare systems' efficacy and quality.

## Author contributions

FiC conceived the research hypothesis. FiC, FB, and AM designed the study. FB, AM, and FrC performed the article screening. FB, AM, FrC, and AZ performed the data extraction and the quality assessment. FiC and WR have shaped the manuscript with input from the entire team (written contributions of single paragraphs). All authors contributed to revise work for important intellectual content, gave the final approval of the version to be published, and agreed on all aspects of the work, especially concerning its accuracy and integrity.

## Conflict of interest

The authors declare that the research was conducted in the absence of any commercial or financial relationships that could be construed as a potential conflict of interest.

## Publisher's note

All claims expressed in this article are solely those of the authors and do not necessarily represent those of their affiliated organizations, or those of the publisher, the editors and the reviewers. Any product that may be evaluated in this article, or claim that may be made by its manufacturer, is not guaranteed or endorsed by the publisher.
